# Lower limb joint loading during high-impact activities: implication for bone health

**DOI:** 10.1093/jbmrpl/ziae119

**Published:** 2024-09-14

**Authors:** Zainab Altai, Claude Fiifi Hayford, Andrew T M Phillips, Jason Moran, Xiaojun Zhai, Bernard X W Liew

**Affiliations:** School of Sport, Rehabilitation and Exercise Sciences, University of Essex, Colchester, CO4 3SQ, United Kingdom; Institute of Public Health and Wellbeing, University of Essex, Colchester, CO4 3SQ, UK; Department of Biomedical Engineering, University of Ghana, Legon, Ghana; Department of Civil and Environmental Engineering, Imperial College London, London, SW7 2ZA, United Kingdom; School of Sport, Rehabilitation and Exercise Sciences, University of Essex, Colchester, CO4 3SQ, United Kingdom; School of Computer Science and Electronic Engineering, University of Essex, Colchester, CO4 3SQ, United Kingdom; School of Sport, Rehabilitation and Exercise Sciences, University of Essex, Colchester, CO4 3SQ, United Kingdom

**Keywords:** high-impact exercises, exercise intensity, lower limb joints, joint contact force, musculoskeletal modeling

## Abstract

Osteoporosis results in low-trauma fractures affecting millions globally, in particular elderly populations. Despite the inclusion of physical activity in fracture prevention strategies, the optimal bone-strengthening exercises remain uncertain, highlighting the need for a deeper understanding of lower limb joint loading dynamics across various exercise types and levels. This study examines lower limb joint loading during high-impact exercises across different intensities. A total of 40 healthy, active participants were recruited (mean ± SD: age of 40.3 ± 13.1 yr; height 1.71 ± 0.08 m; and mass 68.44 ± 11.67 kg). Motion capture data and ground reaction forces of 6 different exercises: a self-selected level of walking, running, countermovement jump, squat jump, unilateral hopping, and bilateral hopping were collected for each participant. Joint reaction forces were estimated using lower body musculoskeletal models developed in OpenSim. Running and hopping increased joint forces compared to walking, notably at the hip (83% and 21%), knee (134% and 94%), and ankle (94% and 77%), while jump exercises reduced hip and ankle loading compared to walking (36% and 19%). Joint loading varied with exercise type and intensity, with running faster increasing forces on all joints, particularly at the hip. Sprinting increased forces at the hip but lowered knee and ankle forces. Higher jumps intensified forces on all joints, while faster hopping reduced forces. The wide variation of lower limb joint loading observed across the exercises tested in this study underscores the importance of implementing diverse exercise routines to optimize overall bone health and strengthen the musculoskeletal structure. Practitioners must therefore ensure that exercise programs include movements that are specifically suitable for their intended purpose.

## Graphical Abstract

**Graphical Abstract:**
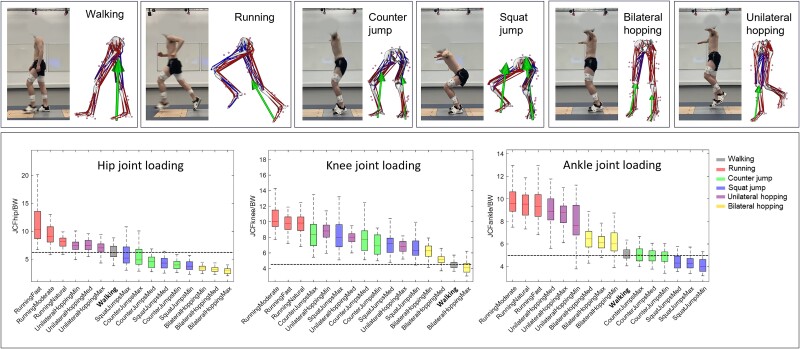

## Introduction

Osteoporosis poses a significant health challenge, particularly among elderly populations. One of the main consequences of osteoporosis is low-impact trauma fractures. Approximately 137 million women and 21 million men worldwide are estimated to face an increased risk of osteoporotic fractures, causing increasingly important public health concerns.^1^ One current strategy aimed at optimizing bone mass to help reduce the risk of osteoporosis-related fractures includes physical activities.^2^ It has been reported that normal walking^3^ is not associated with bone mineral density (BMD) changes in the femoral neck (FN), whereas jogging combined with walking,^4^ running, and jumping^5^^,^^6^ was the most effective in improving bone density and other parameters of bone health. However, the optimal type and intensity of exercises that enhance bone mass are still largely unknown.^7^^,^^8^

The relationship between physical activity, exercise, and bone health is explained by the “mechanostat” theory,^9^ which states that bone adapts its microstructure based on the induced mechanical loadings. These loadings are represented by external factors, including reactionary and inertial forces, as well as internal factors, such as joint contact forces and muscle forces. It is thought that when the imposed force on the bone exceeds a particular threshold, bone formation occurs in favor of bone resorption.^9^ Nevertheless, the optimal force that enhances bone formation is still unknown. According to a meta-analysis by Kistler-Fischbacher et al., a high ground reaction force, of at least 2 times bodyweight, might be required to stimulate an osteogenic effect.^10^ Although ground reaction force has been used by several lab-based motion analysis studies to monitor loading on the musculoskeletal system to understand injury risk, studies have shown that such metrics can mislead our understanding of loading on internal structures.^11^

To predict the osteogenic impact of various exercises, quantitative data on the forces exerted on the joints of the lower limbs are required. These forces generate stresses and strains in the bones, whereby local adaptation of bone microstructure occurs. Recent work reported that joint contact force at the hip is strongly related to the strain distribution in the proximal part of the femur during walking,^12^ running,^13^^,^^14^ and jumping.^14^ However, although there are many benefits to high-impact exercise like jumping, such activities may also carry some potential risks. High-impact movements can exert higher stresses than desired on the joints thus increasing the risk of both acute and overuse injuries. Accordingly, the potential to relate joint contact force at the hip, knee, and ankle during different exercises to stress and strain levels that trigger osteogenesis, without causing injury, could be a major advancement toward optimizing training programs designed to stimulate bone formation.

The “gold standard” method for in vivo measurement of joint contact forces includes using instrumented implants.^15^ However, this method is limited by a small number of subjects in addition to the altered anatomy and physiology of the joint region due to surgery. On the other hand, musculoskeletal modeling based on three-dimensional (3D) motion capture can estimate joint forces comparable to experimental measurements during various physical activities.^16^ Several previous studies have employed musculoskeletal modeling to investigate the effect of different speeds of walking and running on ground reaction forces^17^ and contact forces of various lower limb joints.^18–20^ To the authors’ knowledge, no research has investigated these predictions during different jumping and hopping movements. Studies in which exercise intensity is investigated have focused predominantly on a single joint (hip,^13^^,^^14^^,^^21^ knee,^22^^,^^23^ and ankle^23^) and did not investigate the loading on the lower-limb kinetic chain. This is important as the alteration of loading on one lower limb joint may be associated with changes in the loading of other joints.^24^ One recent study reported a significant increase in the knee joint contact force, but not that of the hip and ankle, with greater walking speed.^25^ Niu et al.^26^ reported that raising the drop height of a jump significantly increased the compressive forces on the hip, knee, and ankle joints. When subjects landed from low (32 cm) and medium heights (52 cm), peak joint contact force in the vertical direction increased,^26^ demonstrating that dropping into a jump from a height produces a different pattern of force production than the traditional form of jumping from a starting position on the ground. In terms of the effect of various exercise types, Pellikaan et al. found that fast walking, running, and unilateral hopping induced significantly higher hip joint contact force than walking at 4 km/h.^14^ However, no information was reported concerning the knee and ankle joints. As aging raises the likelihood of developing knee osteoarthritis, a condition often associated with the potential worsening of other existing health issues,^27^ a comprehensive examination of all 3 joints is warranted.

The present study aimed to quantify hip, knee, and ankle joint loading across various exercise types (walking, running, countermovement jump, squat jump, unilateral hopping, and bilateral hopping) and intensities (low, moderate, and maximum levels). Our objective was to enable practitioners to make more informed selections concerning the types of exercises that can be chosen to protect against acute and chronic injury and the age-related degeneration of joints. Accordingly, we sought to address 2 primary questions: first, which exercises elicit higher loading at the hip, knee, and ankle joints compared to walking at normal speed; and second, how does exercise intensity influence loading at the 3 aforementioned joints? By investigating the type and intensity of exercise that optimally stimulates bone remodeling without compromising joint integrity, this study contributes insights to the development of targeted exercise interventions for individuals at risk of osteoporosis-related issues.

## Materials and methods

### Participants

Forty healthy participants were recruited for the present study, which occurred in the motion laboratory at the University of Essex, UK. Eligible participants must fall within the age range of 18 to 70 yr old, have no lower limb joint replacements, and have not been diagnosed with any serious lower limb injuries within the last year. The final sample included 20 males and 20 females (mean ± SD: age of 40.3 ± 13.1 yr; height 1.71 ± 0.08 m; and mass 68.44 ± 11.67 kg). Ethical approval was obtained from the University of Essex Faculty of Science & Health Ethics Subcommittee (ETH2021-1155). A written consent form was obtained from all participants before participating.

### Experimental setup and protocol

**Figure 1:**
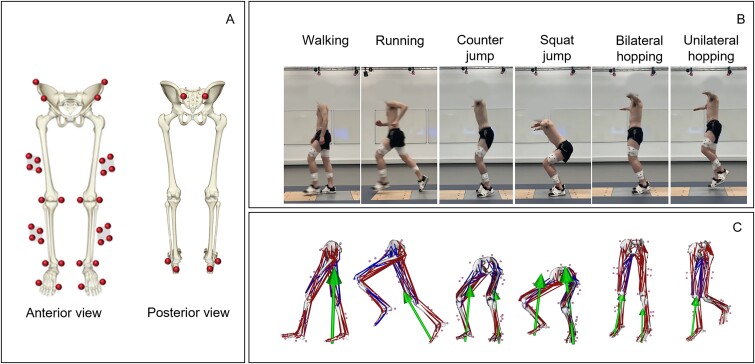
Marker set for motion capture data and musculoskeletal models. Twenty-two individual markers and 4 cluster markers were placed on the bone anatomical landmarks of the lower body (A). Six different exercises; walking, running, countermovement jump, squat jump, unilateral hopping, and bilateral hopping were performed by each participant (B). Scaled musculoskeletal models were developed in OpenSim representing each performed exercise (C).

Thirty-eight retro-reflective markers were attached to the lower extremities of each participant. Twenty-two individual markers were attached to the following anatomical landmarks: left and right superior iliac spines, anterior superior iliac spines and posterior superior iliac spines, medial and lateral femoral condyles, medial and lateral malleoli. On the shoe, rearfoot markers were attached to the calcaneus’s lateral and posterior aspects, while forefoot markers were attached to the first and fifth metatarsals. Furthermore, tracking clusters consisting of 4 markers were attached to the distal lateral aspect of the thigh and the shank (Figure 1). Five electromyography (EMG) sensors (Noraxon USA, 2 kHz) were attached unilaterally to the dominant side of each participant targeting 5 different muscles: gluteus maximus, gluteus medius, rectus femoris, biceps femoris, and soleus following SENIAM guidance. Surface EMG signals were high-pass filtered at 30 Hz using a zero-phase lag, fourth-order Butterworth filter, and rectified. The rectified signals were then low-pass filtered at 10 Hz. The maximum value for each EMG signal was estimated from 3 repetitive trials for each exercise type and intensity, then the envelopes for the EMGs were computed and normalized with their estimated maximum value; this varied from 0 to 1. EMG envelopes were used to verify the musculoskeletal model’s muscle force predictions (Figure SM.1 in supplementary material). Marker trajectories were recorded using 16 3D motion capture cameras (Vicon. Ltd., 200 Hz) and filtered at 18 Hz with a zero-lag second-order low-pass Butterworth. GRFs were collected using 2-floor force plates (Kistler, 2 kHz) positioned side by side (Figure 1B) and filtered at 50 Hz with a zero-lag second-order low-pass Butterworth.

After a verbal description and demonstration of the testing exercises, participants were asked to perform 6 different exercises: walking, running, countermovement jump, squat jump, unilateral hopping, and bilateral hopping. Each exercise, excluding walking, was conducted at 3 self-reported intensity levels: maximum, medium, and minimum. “Running intensity” denotes the speed of running, “jumping intensity” pertains to jump height, and “hopping intensity” refers to the frequency of hopping, reflecting the speed at which the participant performs the hop. For running, participants were asked to run on 20-m runway at 3 targeted speed levels: fast (highest attainable speed), natural (typical jogging pace), and an intermediate medium speed determined collaboratively by the participants and the instructor, taking into consideration the fast and natural running speed. For the countermovement jump, participants started from an upright standing position, briefly countermoved to a self-selected depth, jumped vertically, and then landed with each foot on a separate force plate. For the squat jump, participants started from a squat position at a self-selected depth, held for 2 s, and then jumped. Both countermovement and squat jump were performed at 3 different effort levels: maximum effort (highest attainable height), minimal effort, and medium effort determined collaboratively by the participants and the instructor. For bilateral hopping (with both feet) and unilateral hopping (with dominant side foot), participants were instructed to hop for 10 s at 3 different speeds with distinct beat frequencies (3.0 Hz, 2.6 Hz, and 2.2 Hz, representing maximum effort, medium effort, and minimum effort, respectively).^28^ Beats were played for the participants in advance and were asked to practice until they achieved synchronization. Walking trials were collected at a self-selected speed of normal walking over the same 20-m runway. Participants were allowed to warm up for 2 min before recording the actual trials, and adequate recovery time was provided between trials to prevent fatigue. Three successful trials (whole foot in contact within the force plate area) were collected for each intensity level of each exercise.

### Musculoskeletal modeling

A generic musculoskeletal model (gait2392) developed by Delp et al.^29^ was modified by removing the torso and associated muscles. The modified lower extremity model consisted of 13 body segments, 18 degrees of freedom (DOF), and 86 Hill-type musculotendon actuators. The hip was modeled as a ball and socket joint (3 DOF), while the knee was modeled as a sliding hinge joint (1 DOF rotational joint with translation coupled to the knee flexion angle), and the ankle and subtalar as revolute joints (1 DOF). Each model was scaled to match the subject’s anthropometric characteristics based on marker data of anatomical landmarks at the hip, knee, and ankle during a static trial. The maximal isometric force of each muscle was scaled by the mass of the subject divided by the mass of the generic model raised to the power 2/3.^30^ The maximal isometric force was increased by a factor of 3 for successful simulation of all tasks to allow the generation of high forces required to perform the dynamic movements.^31^ A typical OpenSim simulation pipeline was followed to estimate joint angles and moments using inverse kinematics and inverse dynamics, respectively. Static optimization was used to estimate muscle forces by minimizing the sum of squared muscle activations. Using joint reaction analysis, hip, knee, and ankle contact forces (JCFhip, JCFknee, and JCFankle, respectively) were calculated for the left and right sides. Data analysis for the current study focused on the participants’ dominant side, determined by asking them which foot they used to kick a ball.

### Trial and data processing

Trials were segmented based on type as described in Table 1. Time points used for trial segmentation were defined using Visual 3D (C-Motion Inc.). All trials were then time normalized to 101 time points. Ground reaction forces (GRFs) and joint contact forces (JCFs) were also normalized by the body weight of the participant (BW). Force curves were averaged for the repetitive trials (3 trials per exercise per intensity into 1 average trial per exercise per intensity) for each participant across the 101 time points. Peak values were identified in the averaged curves of each participant, then the means of the peaks were calculated across all participants for each exercise intensity. The averaged curves across all participants of the vertical ground reaction force (vGRF) and the resultant JCFs (JCFhip, JCFknee, and JCFankle) can be found in the Supplemental Material (Figures SM.2– SM.5).

**Table 1:** Time points used to segment trials for each of the 5 tested exercises.

**Exercise type**	**Time point used for trial segmentation**	
	**From**	**To**
**Walking**	Heel strike	Toe off^a^
**Running**	Heel strike	Toe off^a^
**Bilateral hopping**	Foot on the force plate^b^	Foot off force plate^b^
**Unilateral hopping**	Foot on the force plate	Foot off force plate
**Counter jump**	Initial stand (just before take-off)	Lowest position of the pelvis after landing
**Squat jump**	Lowest pelvis position during the squat position (just before take-off)	Lowest position of the pelvis after landing

^a^Of the other foot (step).

^b^Dominant side.

### Statistical analysis

A repeated measure 2-way ANOVA was performed on the peaks of the GRFs and JCFs of all participants to test the global effect of exercise type and exercise intensity compared to walking using the General Linear Model in SPSS. The dependent variable was the JCFs of the hip, knee, ankle, and vGRF, while the independent variables were the various exercise types and intensities. Where significance was found (significance level α = 0.05), a Bonferroni post hoc test was conducted to quantify pairwise differences.

## Results

The means and standard deviations of the peak vGRF, and peak JCFs of the hip, knee, and ankle across all exercise types and intensities are indicated in Table 2. Walking and running speeds, jump height, and stance duration during hopping are also reported in Table 2.

**Table 2:** Mean and SD of the peak vertical ground reaction force and joint contact forces of the hip, knee, and ankle estimated by the musculoskeletal models for the 6 tested exercises.

**Exercise**	**Mean ± SD peak ground reaction forces and joint contact forces normalized by the body weight**				**Mean ± SD Speed (m/s)**	**Mean ± SD Jump height (m)**	**Mean ± SD Stance duration (s)**
	**vGRF**	**JCFhip**	**JCFknee**	**JCFankle**			
**Walking**	1.28 ± 0.09	6.31 ± 1.23	4.50 ± 0.50	5.05 ± 0.53	1.59 ± 0.41	–	–
**Running natural**	2.51 ± 0.26	8.21 ± 1.32	9.70 ± 1.26	9.54 ± 1.43	2.98 ± 0.61	–	–
**Running moderate**	2.62 ± 0.27	9.47 ± 2.17	10.54 ± 2.0	9.79 ± 1.36	4.25 ± 0.59	–	–
**Running fast**	2.62 ± 0.30	11.56 ± 4.1	9.97 ± 1.77	9.51 ± 1.30	5.26 ± 0.83	–	–
**Squat jumps min**	1.05 ± 0.15	4.06 ± 1.33	6.65 ± 1.38	4.11 ± 0.66	–	0.21 ± 0.06	–
**Squat jumps med**	1.08 ± 0.14	4.47 ± 1.45	7.13 ± 1.53	4.44 ± 0.65	–	0.26 ± 0.08	–
**Squat jumps max**	1.03 ± 0.11	5.83 ± 2.21	8.32 ± 2.05	4.32 ± 0.59	–	0.32 ± 0.09	–
**Counter jumps min**	1.22 ± 0.18	4.18 ± 1.52	7.26 ± 1.69	4.92 ± 0.67	–	0.23 ± 0.05	–
**Counter jumps med**	1.18 ± 0.13	4.59 ± 1.44	7.80 ± 1.82	4.92 ± 0.58	–	0.28 ± 0.07	–
**Counter jumps max**	1.19 ± 0.14	5.63 ± 2.38	8.88 ± 2.59	5.04 ± 0.64	–	0.33 ± 0.08	–
**Unilateral hopping min**	2.36 ± 0.27	7.65 ± 1.43	8.74 ± 1.48	8.04 ± 1.64	–	–	0.31 ± 0.05
**Unilateral hopping med**	2.59 ± 0.25	7.56 ± 1.46	8.02 ± 1.13	8.96 ± 1.58	–	–	0.28 ± 0.06
**Unilateral hopping max**	2.59 ± 0.22	6.98 ± 1.14	6.84 ± 0.92	8.72 ± 1.23	–	–	0.25 ± 0.06
**Bilateral hopping min**	1.63 ± 0.29	3.50 ± 0.74	6.39 ± 1.14	6.22 ± 1.35	–	–	0.25 ± 0.04
**Bilateral hopping med**	1.76 ± 0.24	3.18 ± 0.57	5.21 ± 0.76	6.55 ± 1.15	–	–	0.21 ± 0.03
**Bilateral hopping max**	1.73 ± 0.22	2.93 ± 0.76	4.20 ± 0.86	6.27 ± 0.96	–	–	0.19 ± 0.02

Abbreviations: JCFankle, ankle joint contact force; JCFhip, hip joint contact force; JCFknee, knee joint contact force; Max, maximum; Min, minimum; Med, medium; SD, standard deviation; vGRF, vertical ground reaction force.

### Exercise type

**Figure 2:**
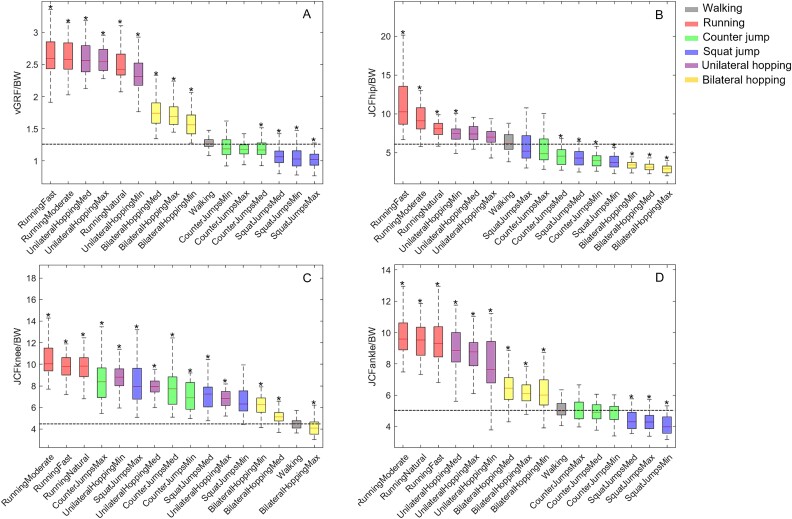
Box plot of large significant difference of peak vertical ground reaction force (A), resultant joint contact forces of the hip (B), knee (C), and ankle (D) expressed in body weight of each participant (BW) compared to walking indicated by the horizontal line. Asterisks denote the exercises with significant difference (^*^p < .05) compared to walking. Peak forces are ranked from left to right for the highest to the lowest estimated values for all tested exercises.

All tested exercises were ranked based on the average peak vGRF (Figure 2A), and peak of JCFhip (Figure 2B), JCFknee (Figure 2C), and JCFankle (Figure 2D). Exercises with a significant difference (*p* < .05) compared to walking with self-selected speed were marked with an asterisk. The estimates, lower/upper limits, and *p*-values as well as the results from the ANOVA are reported in the Supplemental Material (Table SM.1).

### Vertical ground reaction force

Running, unilateral hopping, and bilateral hopping imposed higher vGRF compared to walking by up to 105%, 103%, and 38%, respectively. Both countermovement jump and squat jump were lower than walking by 8% and 20%, respectively.

### Peak joint contact forces

Running and unilateral hopping imposed higher peak forces on the hip joint compared to walking, with increases of up to 83% and 21%, respectively. Conversely, peak forces on the hip joint decreased by 53%, 36%, and 34% during bilateral hopping, squat jumps, and countermovement jumps, respectively.

All exercises resulted in higher peak knee JCFs compared to walking. Running induced the highest peak force, increasing by up to 134%, followed by countermovement jumps at 97%, unilateral hopping at 94%, and squat jumps at 85%, with the lowest increase during bilateral hopping at 42% higher than walking.

Similar to the hip joint, the ankle joint experienced increased peak loading during running (94%), unilateral hopping (77%), and bilateral hopping (30%) compared to walking. Conversely, both squat jumps and countermovement jumps imposed lower peak forces on the ankle joint than walking, with reductions of 19% and 3%, respectively.

### Exercise intensity

Overall, the impact of exercise intensity on the vGRF was negligible across all exercises with only a minor increase observed when hopping at a higher frequency. A clear effect was observed on JCFs (supplementary material Figure SM.6). Increasing running speed from jogging to a moderate speed resulted in increased forces across all joints. However, sprinting at the highest capacity exhibited a considerably higher force at the hip joint combined with decreased forces on the knee and ankle joints. Increased vertical jump amplitude caused an increase in forces experienced at the hip, knee, and ankle joints. Conversely, increased hopping frequency results in a reduction in forces exerted across all joints.

## Discussion

The current study explores the effect of high-impact exercises at different intensity levels on hip, knee, and ankle joint loadings. The aim was to evaluate the types and intensity of exercises that may support its use when designing physical training to stimulate bone tissue formation. Running and hopping at all tested intensities resulted in significantly higher JCFs and vGRFs in the 3 joints compared to normal walking speed. Hip joint load was higher than the knee and ankle during walking, while knee joint load was higher during running and jumping, and ankle load was higher during hopping.

The range of JCFs of the hip, knee, and ankle joints estimated by the current study is comparable to previous studies.^13^^,^^14^^,^^19^^,^^32–34^ Prior simulation studies reported hip joint forces during slow to fast runs to be 7.5–10.0 BW^13^^,^^19^ (speed range of 0.8–3.6 m/s). This is comparable to the estimated 8.2–11.6 BW in the current study. Knee joint forces were previously reported as being 7.8–12.0 BW at 4.36 m/s^19^^,^^32^ while our prediction stood at 9.7–10.5 BW at 2.98–4.25 m/s. Furthermore, ankle joint forces ranged from 11.9 BW to 13 BW at 4.36 m/s^19^^,^^33^ compared to our prediction of around 10.0 BW. Some of our estimated forces were slightly higher than the aforementioned studies.^13^^,^^19^^,^^33^ One potential reason for this could be due to our cohort comprising healthy, active individuals who exercised at least 3 times per week. This is supported by the higher running speed ranges observed in the current study cohort (2.98–5.25 m/s) compared to those studies.^13^^,^^19^^,^^33^ Another reason for this finding could be related to the use of the static optimization technique to estimate muscle forces in the utilized musculoskeletal models. Static optimization methods were previously reported to likely overestimate lower-limb JCFs during vigorous gait tasks.^19^ However, static optimization solutions have also been observed to be practically equivalent to dynamic solutions.^36^

In terms of hopping and jumping, despite the limited available information in the literature, our results align with the existing data. Pellikaan et al. reported a hip joint contact force range of 6.0–7.57 BW during self-selected unilateral hopping.^14^ The study included post-menopausal elderly women, potentially leading to lower reported values compared to our study. Joint contact forces during countermovement jump performed by athletic males were reported to be 5.5–8.4 BW, 6.9–9.0 BW, and 8.9–10.0 BW at 0.38 m jump height^37^ compared to our predictions 4.2–5.6 BW, 7.3–8.9 BW, and 4.9–5.0 BW at jump height range 0.23–0.33 m for hip, knee, and ankle joints, respectively. Knee joint contact force during squat jumps was reported to have an average peak of 7.07 BW^35^ compared to the current study prediction 6.65 BW– 8.32BW at jump height range 0.21–0.32 m.

The current study assumes normal walking, which is a low-impact daily activity, as the baseline against which each exercise is evaluated in terms of its potential for promoting beneficial changes in bone structure and density.^3^^,^^14^^,^^38^ Running and hopping involve brief periods of weightlessness followed by forceful ground impacts and joint loading, inducing a more significant osteogenic response chronically over time. These results are partly in agreement with several clinical trials and simulation studies. Fast-walking,^4^^,^^39^ running,^14^ and hopping^14^^,^^40^ intervention programs were previously reported to preserve FN BMD in an elderly population. Interestingly, our results suggest a limited effect of countermovement jump and squat jump exercises on this outcome even when jumps are performed with maximal effort, in particular at the hip and ankle joint regions at which peak joint loading was found to be lower than that observed during walking. In all the tested exercises, the shank, thigh, and trunk are oriented closer to the vertical than the horizontal direction throughout the stance phase. These orientations create longer moment arms for horizontal forces and shorter moment arms for vertical forces. As the body moves forward during forward locomotion, like walking and running, the moment arm of the horizontal forces has been shown to increase by up to 3.8 times compared to those for vertical forces, thus generating relatively high torques on the body segments. In contrast, during vertical motions like jumping and hopping, the upright posture directs the GRFs relatively close to the joint centers, reducing the moment arms of the force at the joints. Accordingly, it appears, based on our work, that jumping and bilateral hopping exercises used in this study seemed to be less suited for increasing BMD. Despite jump training being previously reported as a high-risk activity for lower extremity joint overloading,^41^ Sen et al.^42^ observed that high-impact programs of bilateral jumping and jump rope exercises could improve functional mobility albeit without any significant changes in BMD values at the femoral neck and lumber spine sites. However, the effectiveness of jumping exercises in promoting BMD improvements can be influenced by various factors, including the frequency, duration, and intensity of the jumps, as well as the overall duration of the training program.^43^ Moreover, individual variations in participants’ baseline BMD levels, adherence to the training regimen, and other lifestyle factors may also play a role in the effectiveness of the exercise in promoting BMD.^44^ Therefore, it is crucial to consider these factors, which have not been fully explored in the current study, when interpreting its findings regarding the impact of jumping on BMD.

The joint that experienced the largest contact forces in the current study differed between exercises. When individualizing the effect of each exercise on each joint, the estimated values of JCFs during running were highest at the knee, followed by the hip, and then the ankle with loads at all joints being higher than in walking. Jumping induced a higher loading at the knee than at the hip and ankle, while hopping loaded the ankle joint the most, followed by the knee and then the hip (load at the knee and ankle were higher than walking). Our study suggests that exercises improve bone strength in a site-specific pattern. While running might enhance bone formation of all lower limb joints, jumping may improve bone formation at the knee joint while also having a limited effect on the hip and ankle sites. Hopping appears to have a greater effect on the ankle joint than on the knee joint but seems to have little effect on the hip joint. Therefore, a well-rounded exercise program for the maintenance of BMD should include a combination of these 3 different exercise types to ensure that bone health is enhanced specifically at each site. It is noteworthy, however, that each high-impact exercise was investigated only in isolation and so the combined, potentially additive, effects of these exercises were not assessed. These exercises contribute to both acute and chronic loading, albeit in different ways. For instance, walking provides a consistent, moderate level of impact over an extended period, contributing to chronic loading, while hopping may offer a more intense, short-term impact, contributing to acute loading. This is an important factor for clinicians to consider as a conventional exercise program may be structured to incorporate a variety of high-impact exercises at varying intensities, alongside complementary strength training to provide the most versatile stimulus as possible.^8^

When considering exercise intensity, hip, knee, and ankle JCFs increased with higher jumping height but decreased with increased hopping frequency. Although there is limited information in the literature regarding the relationship between lower limb joint loading and the intensity level of hopping and jumping exercises, this relationship can be partially explained by the amplitude of muscle activation and the cumulative loading on joints during specific exercises. Previous studies have indicated that increased eccentric strength plays a role in enhancing countermovement jump height.^45^ This is attributed to the correlation between eccentric strength during knee extension and squatting exercises and the resulting jump height. On the other hand, one would typically experience a lower jump height at a higher hopping frequency. Increasing hopping frequency is combined with a decrease in hip flexion, knee flexion, and ankle dorsiflexion, which in turn results in an increase in the stiffness of lower limb joints^46^ and reduced muscle activations when measured by EMG.^47^ When running was performed at maximum effort (sprinting at 5.26 m/s), the increased force at the hip joint was offset by decreased force at the knee and ankle joints. Large hip muscles, such as the gluteal muscles, become highly active when running rapidly to counteract joint moments and generate higher forces at the hip.^48^ In support of our findings, running at a slower speed (2.23 m/s) was previously reported to increase accumulated loads at the knee as compared to faster running (4.38 m/s).^49^ Peterson et al. explained that the primary reason for the increase in cumulative load at slower speeds is an increase in the number of strides needed to cover the same distance.^49^

It is worth noting that the impact of exercise intensity on the vGRF was negligible as compared to JCFs across the exercises as shown in Table 1. Therefore, the use of GRFs may not serve as an appropriate predictor of peak skeletal loading at specific joints and so should not be used to assess the intensity of an exercise regimen in isolation.^11^ The discrepancies between joint loads and ground reaction forces suggest that other factors, such as muscle activation levels also play an important role in joint loading.^13^ This is related to muscles exerting considerable forces to equilibrate the external moments caused by GRFs during a given motion, serving as protective mechanism against damage to the surrounding non-contractile tissue.^50^

The present results should be interpreted with caution owing to certain limitations. Our study employed the static optimization technique. This technique does not account for the temporal aspects of muscle activation and coordination, which are important for dynamic tasks such as those in this study and can lead to erroneous predictions of muscle force.^51^ Future studies may investigate JCFs prediction for high-impact activities using subject-specific MRI-based musculoskeletal models^52^ and dynamic optimization or forward dynamic simulations. Nonetheless, static optimization has been found to adequately replicate muscle activation patterns during walking^36^ and hopping^53^ even though the magnitudes of the produced forces remain unverified due to the impracticality of in vivo data acquirement. Furthermore, our estimated muscle force patterns have been evaluated against the measured EMG signals (Supplementary material Figure SM.2). Another limitation is that passive soft tissues can be engaged during movements such as at the extremes of joint range of motion, and not accounting for their contribution to joint loading can lead to the underestimation of JCFs.^54^ Other factors related to the study cohort included healthy, highly active individuals of both sexes, spanning different age groups. Peak joint loading from young adults cannot be generalized to elderly populations,^55^ and joint kinematics differ between males and females.^56^ Therefore, the current results should not be interpreted to age- or sex-specific populations. Further studies can address the current shortfall in the literature.

### Conclusion

The investigation of lower limb joint performance during high-impact activities in this study contributes to the understanding of how much exercise influences joint loading, offering implications for optimizing exercise regimens to enhance bone health. Based on the results of this investigation, we recommend that coaches should expose trainees to a diverse and varied range of exercises, which subject the lower limb joints to forces of different types and magnitude. This is reflective of the wide variation in vGRF and JCFs observed across each of the different exercises in this study. As these exercises are reflective of those used functionally throughout a typical person’s life, they represent the types of tasks that place strain on the musculoskeletal structures. Accordingly, building strength and resilience to these strains seems a prudent approach to both preserve and increase bone health. Coaches should consider such exercises in the amount that is suitable to a trainee’s level of experience and exercise tolerance though this is likely to vary significantly between populations. Moreover, the current study findings are crucial not only for optimizing exercise regimens to enhance bone health but also for various other aspects, such as designing risk prevention and rehabilitation programs, developing prosthetics, and analyzing sports or occupational activities. Future studies should prioritize establishing population-specific thresholds for the amount of exercise required to enhance bone health. Additionally, there is a need for research to investigate the stress and strain effects on joints, aiming to deepen our understanding of how joint forces generated by different exercise types and intensities might stimulate bone formation.

## Supplementary Material

SupplementaryMaterial_clean_ziae119

## Data Availability

The datasets for this study can be found in the GitHub https://github.com/ZainabAltai/Lower-limb-joint-loading-during-high-impact-activates-.
