# Supplementary material for: Lower limb joint loading during high-impact activities: implication for bone health
Source: JBMR Plus. 2024 Sep 14;8(11):ziae119. doi: 10.1093/jbmrpl/ziae119 (PMC11481284; doi:10.1093/jbmrpl/ziae119)
Supplement: SupplementaryMaterial_clean_ziae119 [file supplementarymaterial_clean_ziae119.docx]

Supplementary Material

Lower limb joint Loading during high-impact activities: implication for bone health

Zainab Altai^a,b*^, Claude Fiifi Hayford^c^, Andrew Phillips^d^, Jason Moran^a^, Xiaojun Zhai^e^, Bernard X.W. Liew^a^

^a^School of Sport, Rehabilitation and Exercise Sciences, University of Essex, Colchester CO4 3SQ, United Kingdom

^b^Institute of Public Health and Wellbeing, University of Essex, Colchester CO4 3SQ, United Kingdom

^c^Department of Biomedical Engineering, University of Ghana, Legon, Ghana

^d^Department of Civil and Environmental Engineering, Imperial College London, London (SW7 2ZA), United Kingdom

^e^School of Computer Science and Electronic Engineering, University of Essex, Colchester CO4 3SQ, United Kingdom

*** Correspondence:**Zainab Altai
[za21920@essex.ac.uk](mailto:za21920@essex.ac.uk); zainabaltai0@gmail.com

| **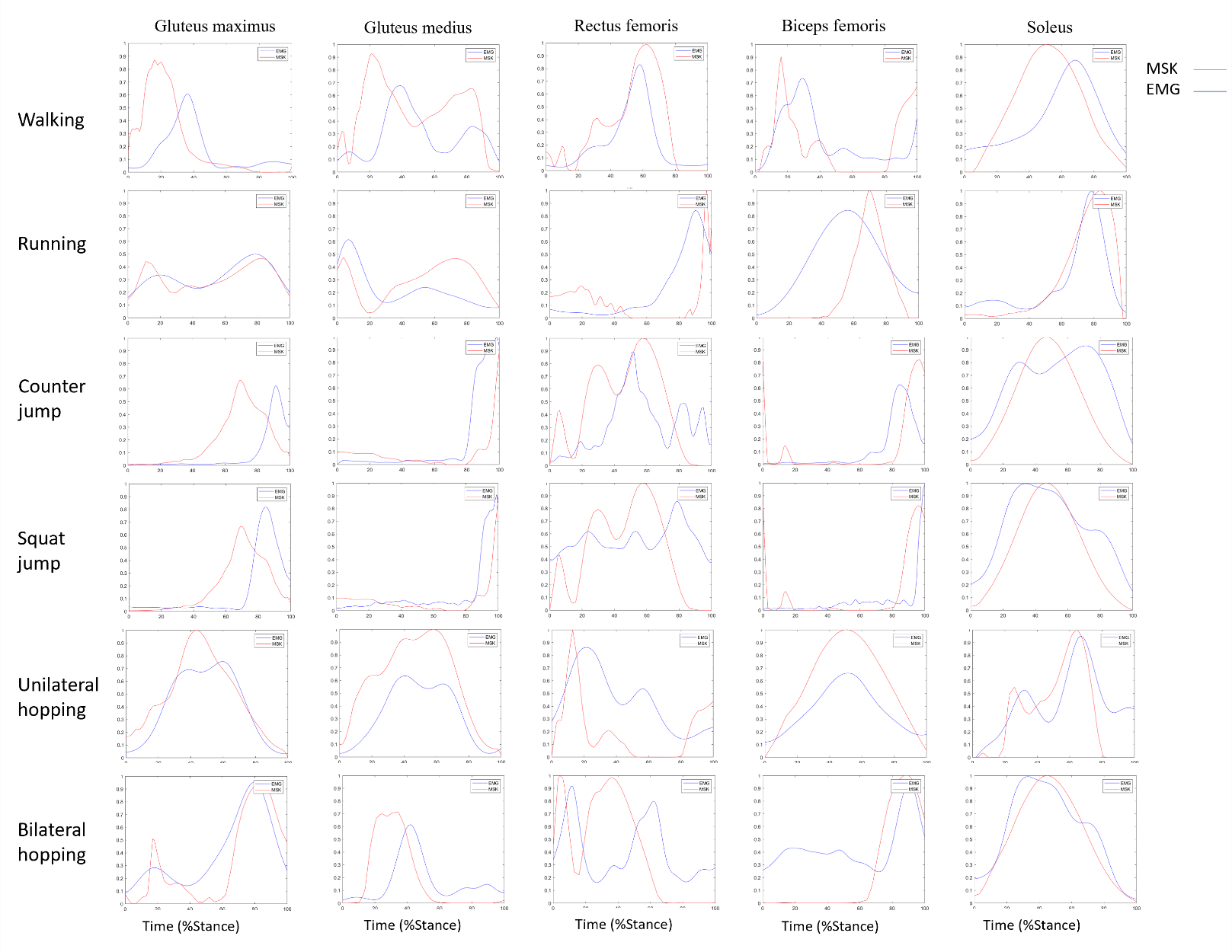** |
| --- |
| **Fig. SM.1.** Normalized muscular activations obtained by the musculoskeletal models (MSK) vs. normalized EMG measurements during walking, running, counter movement jump, squat jump, unilateral hopping, and bilateral hopping of a representative participant. |

| 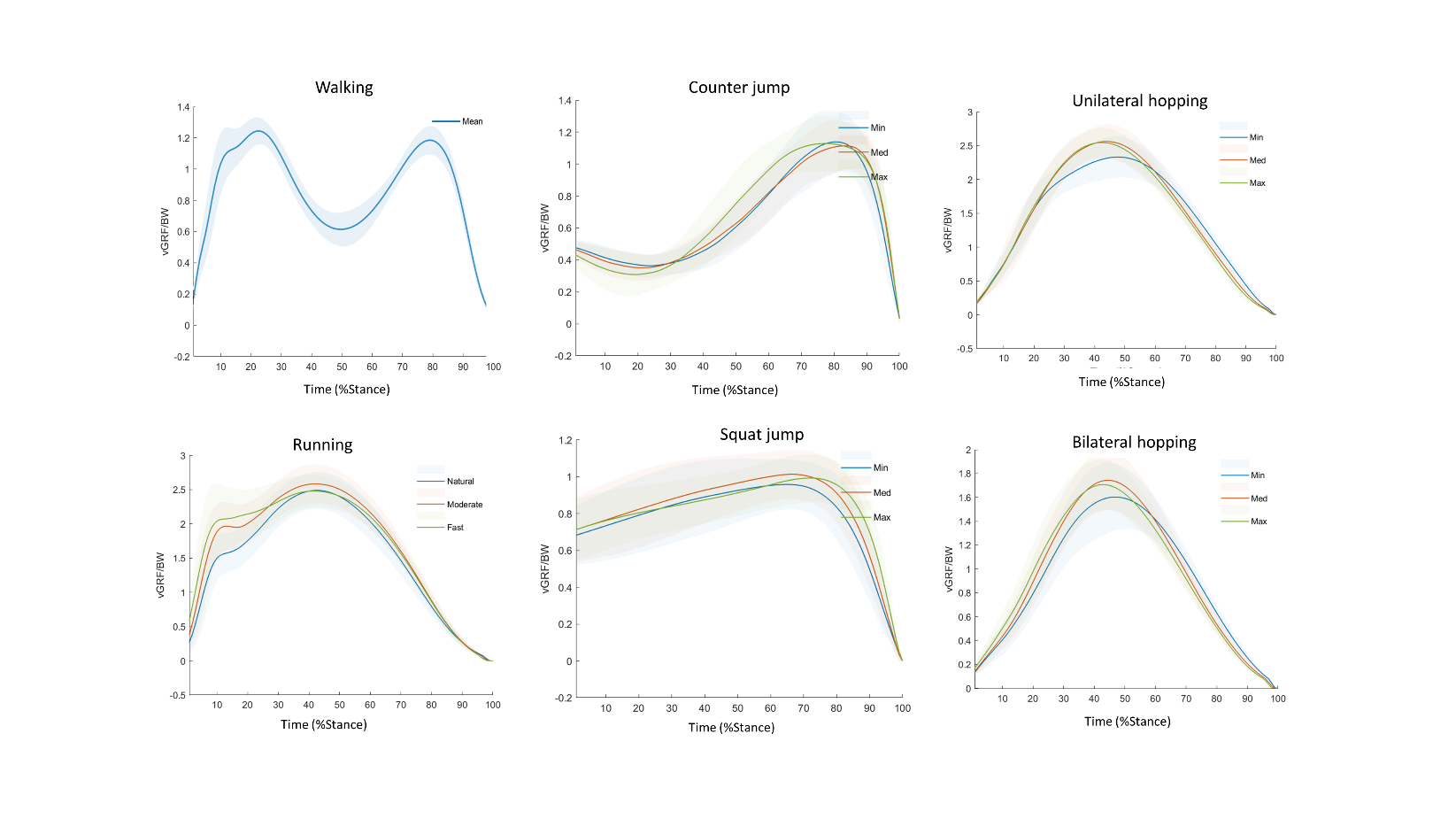 |
| --- |
| **Fig. SM.2.** Mean curves of the vertical ground reaction forces predicted by the musculoskeletal models and normalized by the body weight (vGRF/BW) for all participants. Min, Med, and Max are the effort, minimum, medium, and maximum effort, respectively, for counter movement jump, squat jump, unilateral hopping, and bilateral hopping. Natural, Moderate, and Fast are the minimum, medium, and maximum effort, respectively, for running. Only one level (self-selected speed) was performed by the participate for walking. |

| 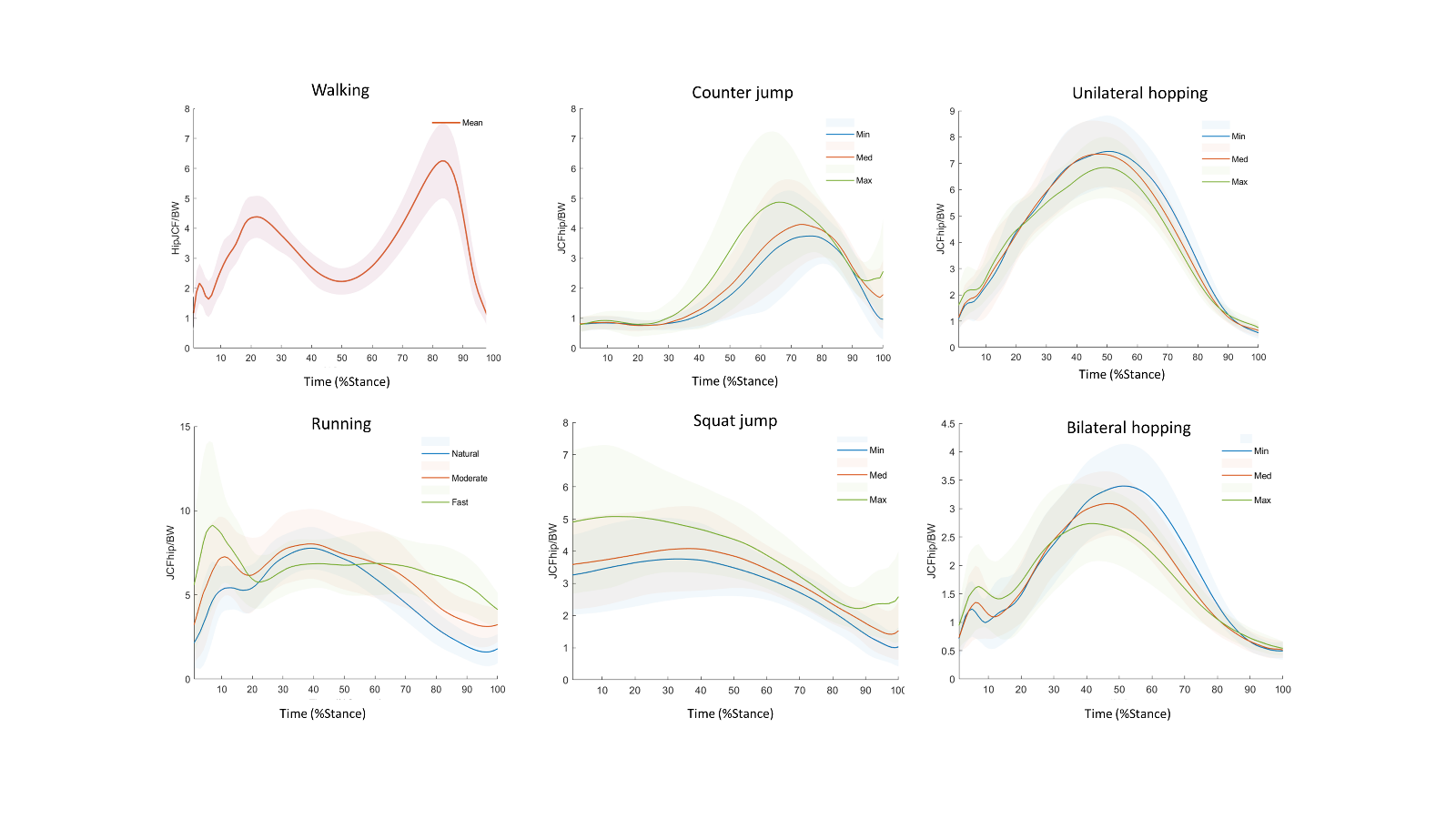 |
| --- |
| **Fig. SM.3.** Mean curves of the resultant hip joint reaction forces predicted by the musculoskeletal models and normalized by the body weight (JCFhip/BW) for all participants. Min, Med, and Max are the effort, minimum, medium, and maximum effort, respectively, for counter movement jump, squat jump, unilateral hopping, and bilateral hopping. Natural, Moderate, and Fast are the minimum, medium, and maximum effort, respectively, for running. Only one level (self-selected speed) was performed by the participate for walking. |
| 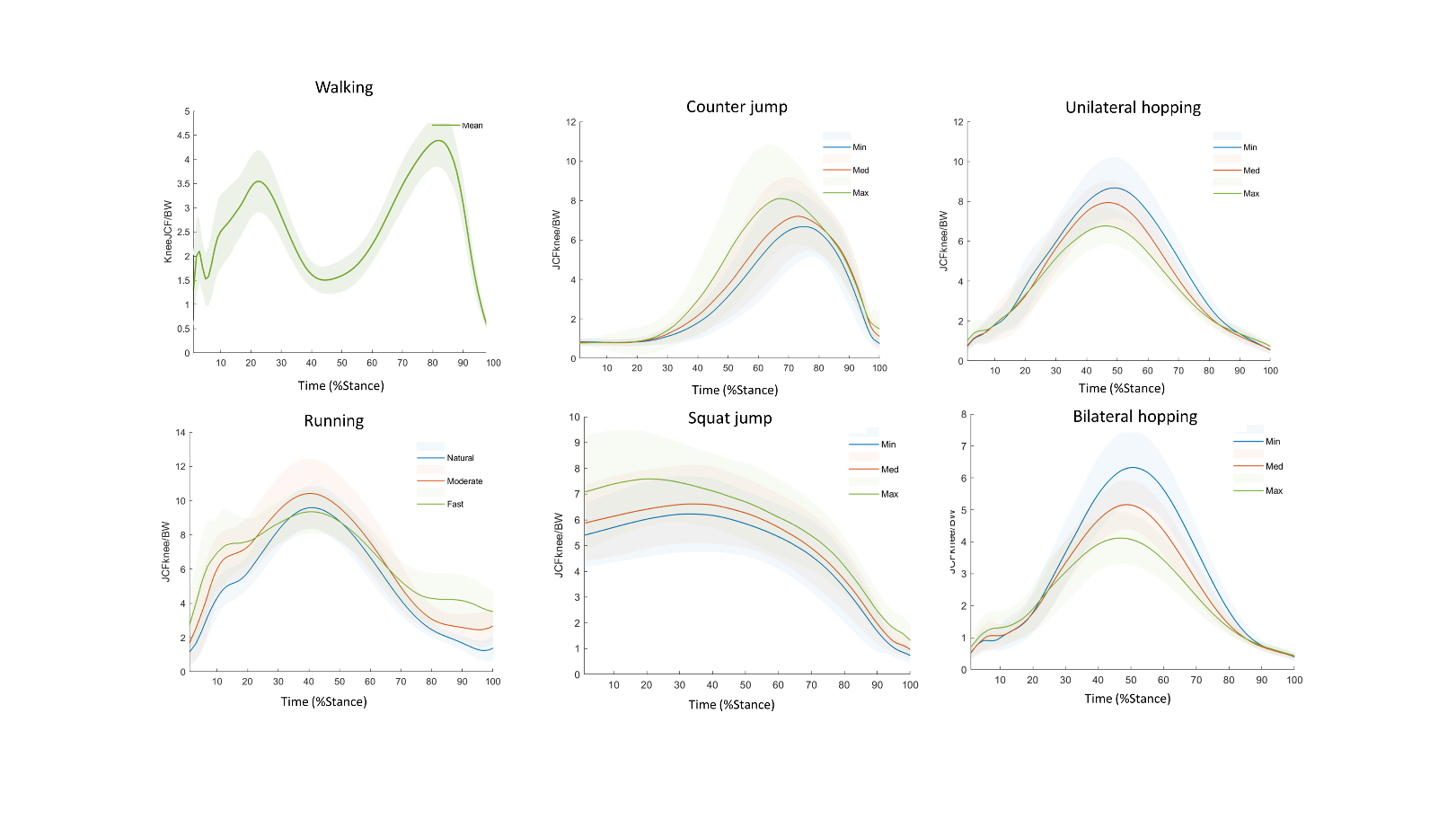 |
| **Fig. SM.4.** Mean curves of the resultant knee joint reaction forces predicted by the musculoskeletal models and normalized by the body weight (JCFknee/BW) for all participants. Min, Med, and Max are the effort, minimum, medium, and maximum effort, respectively, for counter movement jump, squat jump, unilateral hopping, and bilateral hopping. Natural, Moderate, and Fast are the minimum, medium, and maximum effort, respectively, for running. Only one level (self-selected speed) was performed by the participate for walking. |

| 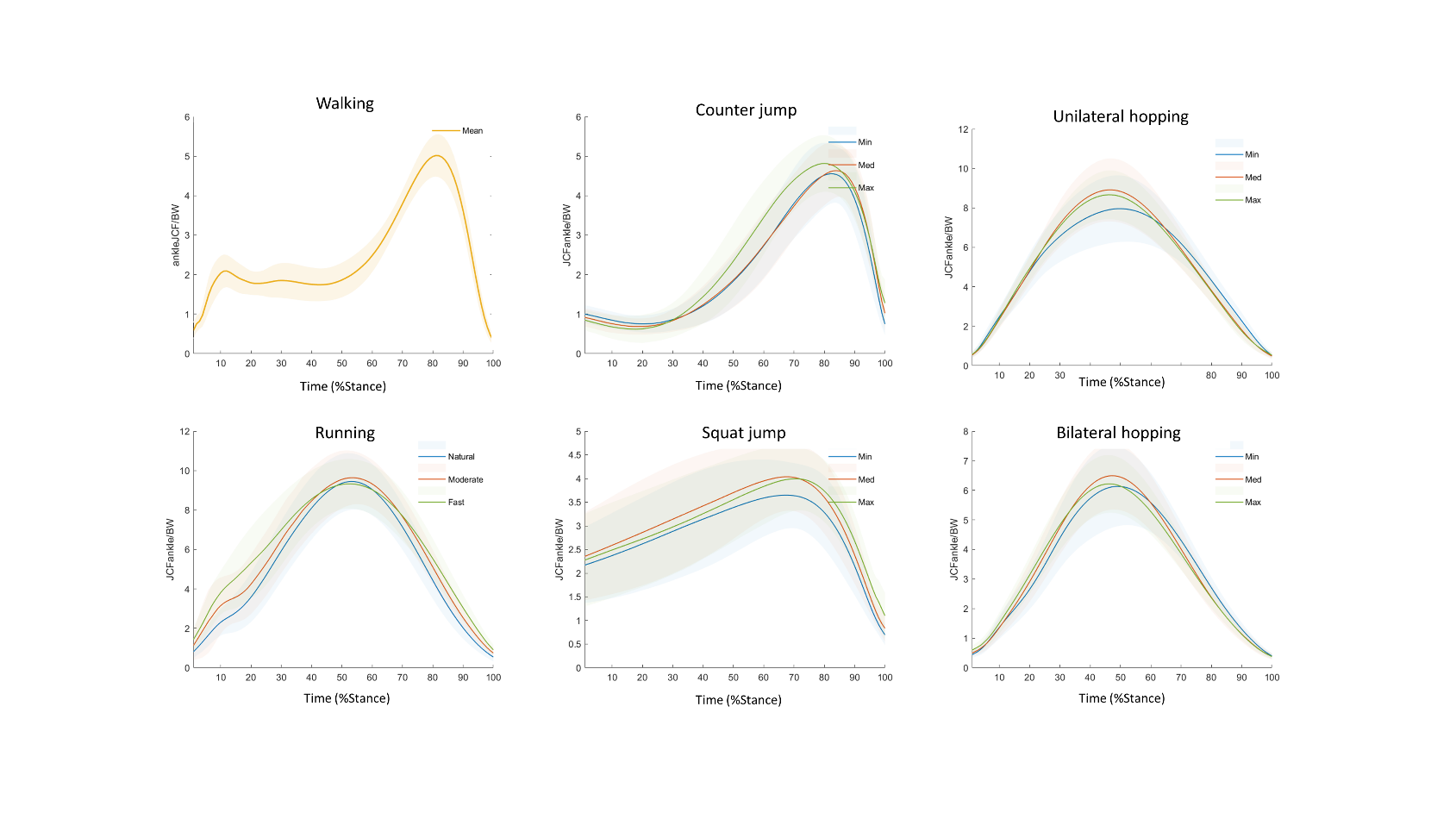 |
| --- |
| **Fig. SM.5.** Mean curves of the resultant ankle joint reaction forces predicted by the musculoskeletal models and normalized by the body weight (JCFankle/BW) for all participants. Min, Med, and Max are minimum, medium, and maximum effort, respectively, for counter movement jump, squat jump, unilateral hopping, and bilateral hopping. Natural, Moderate, and Fast are the minimum, medium, and maximum effort, respectively, for running. Only one level (self-selected speed) was performed by the participate for walking. |

| **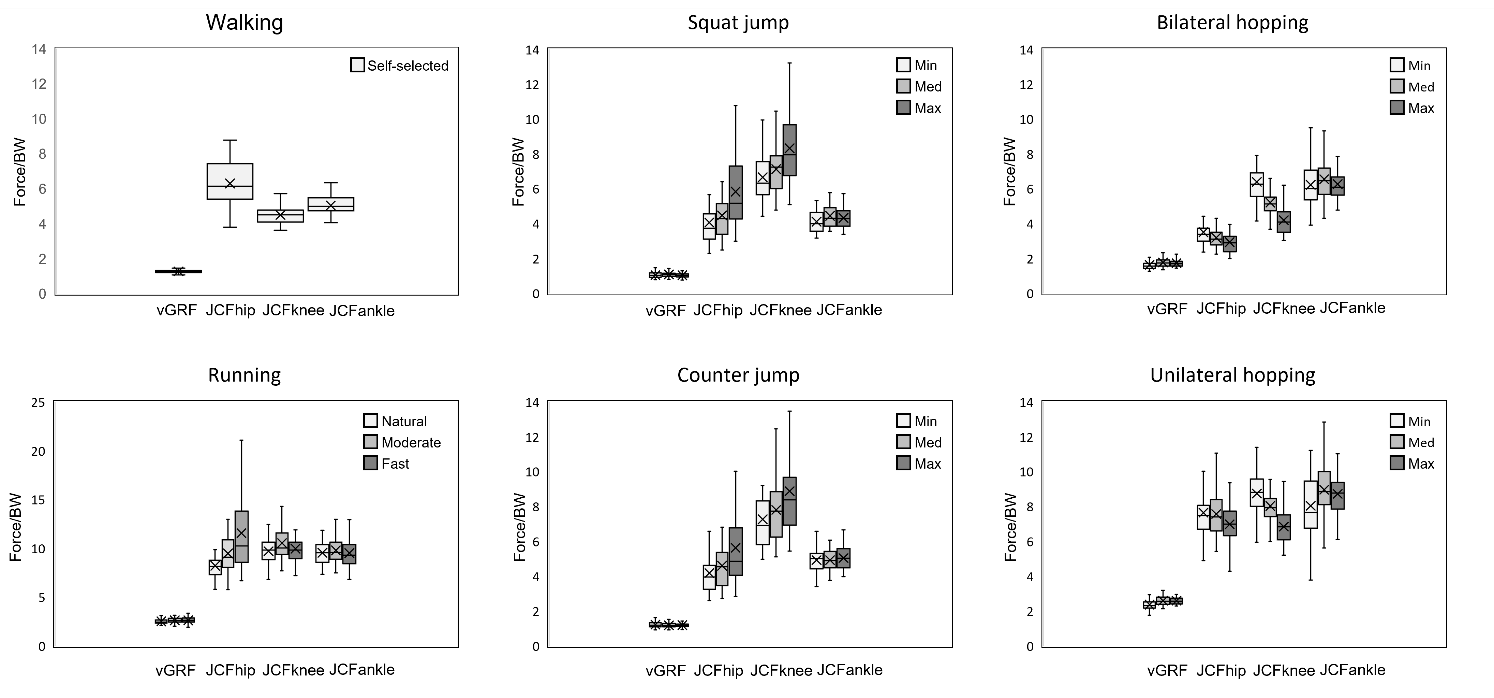** |
| --- |
| **Fig. SM.6.** Distribution of peak vGRF and peak JCFs during walking, running, squat jump, counter movement jump, unilateral hopping, and bilateral hopping and at three different exercise intensities. vGRF ground reaction force; JCF contact force at hip, knee, and ankle. |

**Table SM.1.** The estimates, lower/upper limits, and p-values as well as the results from the ANOVA test on the vertical ground reaction force and joint contact force of the hip, knee, and ankle. Exercises with a significant difference (p < .05) compared to walking are marked with an asterisk.

|  | **Exercise** | **Mean Difference (walking-exercise)** | **Std. Error** | **P value** | **95% Confidence Interval for Difference** | |
| --- | --- | --- | --- | --- | --- | --- |
|  |  |  |  |  | **Lower Bound** | **Upper Bound** |
| vGRF | RunningNatural | -1.230^*^ | .043 | .000 | -1.396 | -1.064 |
|  | RunningModerate | -1.337^*^ | .044 | .000 | -1.508 | -1.166 |
|  | RunningFast | -1.338^*^ | .049 | .000 | -1.527 | -1.149 |
|  | SquatJumpsMin | .233^*^ | .029 | .000 | .121 | .346 |
|  | SquatJumpsMed | .200^*^ | .027 | .000 | .096 | .305 |
|  | SquatJumpsMax | .251^*^ | .026 | .000 | .151 | .351 |
|  | CounterJumpsMin | .056 | .030 | 1.000 | -.059 | .171 |
|  | CounterJumpsMed | .096^*^ | .025 | .049 | .000 | .192 |
|  | CounterJumpsMax | .088 | .025 | .111 | -.007 | .184 |
|  | UnilateralHoppingMin | -1.082^*^ | .049 | .000 | -1.273 | -.891 |
|  | UnilateralHoppingMed | -1.312^*^ | .046 | .000 | -1.488 | -1.136 |
|  | UnilateralHoppingMax | -1.308^*^ | .038 | .000 | -1.456 | -1.159 |
|  | BilateralHoppingMin | -.347^*^ | .049 | .000 | -.535 | -.158 |
|  | BilateralHoppingMed | -.483^*^ | .043 | .000 | -.649 | -.318 |
|  | BilateralHoppingMax | -.452^*^ | .035 | .000 | -.586 | -.319 |
| JCFhip | RunningNatural | -1.900* | .240 | .000 | -2.828 | -.972 |
|  | RunningModerate | -3.163* | .400 | .000 | -4.705 | -1.620 |
|  | RunningFast | -5.249* | .652 | .000 | -7.765 | -2.733 |
|  | SquatJumpsMin | 2.250* | .300 | .000 | 1.093 | 3.407 |
|  | SquatJumpsMed | 1.839* | .309 | .000 | .648 | 3.030 |
|  | SquatJumpsMax | .477 | .436 | 1.000 | -1.206 | 2.160 |
|  | CounterJumpsMin | 2.131* | .340 | .000 | .820 | 3.442 |
|  | CounterJumpsMed | 1.715* | .329 | .001 | .447 | 2.984 |
|  | CounterJumpsMax | .680 | .453 | 1.000 | -1.068 | 2.428 |
|  | UnilateralHoppingMin | -1.339* | .281 | .003 | -2.422 | -.255 |
|  | UnilateralHoppingMed | -1.252* | .284 | .010 | -2.348 | -.155 |
|  | UnilateralHoppingMax | -.668 | .238 | .943 | -1.588 | .252 |
|  | BilateralHoppingMin | 2.807* | .201 | .000 | 2.030 | 3.584 |
|  | BilateralHoppingMed | 3.125* | .190 | .000 | 2.393 | 3.858 |
|  | BilateralHoppingMax | 3.375* | .203 | .000 | 2.592 | 4.159 |
| JCFknee | RunningNatural | -5.194* | .225 | .000 | -6.061 | -4.327 |
|  | RunningModerate | -6.034* | .340 | .000 | -7.346 | -4.723 |
|  | RunningFast | -5.460* | .302 | .000 | -6.627 | -4.293 |
|  | SquatJumpsMin | -2.149* | .245 | .000 | -3.093 | -1.204 |
|  | SquatJumpsMed | -2.623* | .263 | .000 | -3.638 | -1.608 |
|  | SquatJumpsMax | -3.811* | .348 | .000 | -5.154 | -2.468 |
|  | CounterJumpsMin | -2.759* | .292 | .000 | -3.885 | -1.634 |
|  | CounterJumpsMed | -3.295* | .307 | .000 | -4.482 | -2.109 |
|  | CounterJumpsMax | -4.370* | .425 | .000 | -6.010 | -2.731 |
|  | UnilateralHoppingMin | -4.237* | .251 | .000 | -5.206 | -3.267 |
|  | UnilateralHoppingMed | -3.519* | .201 | .000 | -4.295 | -2.744 |
|  | UnilateralHoppingMax | -2.334* | .168 | .000 | -2.981 | -1.687 |
|  | BilateralHoppingMin | -1.888* | .196 | .000 | -2.642 | -1.133 |
|  | BilateralHoppingMed | -.703* | .141 | .002 | -1.247 | -.158 |
|  | BilateralHoppingMax | .308 | .142 | 1.000 | -.241 | .858 |
| JCFankle | RunningNatural | -4.491* | .220 | .000 | -5.339 | -3.643 |
|  | RunningModerate | -4.744* | .220 | .000 | -5.591 | -3.897 |
|  | RunningFast | -4.458* | .199 | .000 | -5.224 | -3.692 |
|  | SquatJumpsMin | .939* | .142 | .000 | .390 | 1.488 |
|  | SquatJumpsMed | .605* | .134 | .007 | .088 | 1.121 |
|  | SquatJumpsMax | .729* | .127 | .000 | .238 | 1.220 |
|  | CounterJumpsMin | .130 | .127 | 1.000 | -.361 | .621 |
|  | CounterJumpsMed | .129 | .120 | 1.000 | -.336 | .593 |
|  | CounterJumpsMax | .005 | .118 | 1.000 | -.450 | .460 |
|  | UnilateralHoppingMin | -2.988* | .263 | .000 | -4.001 | -1.975 |
|  | UnilateralHoppingMed | -3.912* | .239 | .000 | -4.833 | -2.990 |
|  | UnilateralHoppingMax | -3.672* | .180 | .000 | -4.366 | -2.978 |
|  | BilateralHoppingMin | -1.171* | .227 | .001 | -2.047 | -.296 |
|  | BilateralHoppingMed | -1.500* | .195 | .000 | -2.254 | -.746 |
|  | BilateralHoppingMax | -1.217* | .156 | .000 | -1.819 | -.616 |
